# Lifetime Prevalence and Comorbidity of Mental Disorders in the Two-wave 2002–2018 Canadian Armed Forces Members and Veterans Mental Health Follow-up Survey (CAFVMHS): Prévalence et Comorbidité de Durée de vie Des Troubles Mentaux Dans l’Enquête de Suivi Sur la Santé Mentale Auprès des Membres des Forces Armées Canadiennes et Des ex-Militaires (ESSMFACM) en Deux Cycles de 2002 à 2018

**DOI:** 10.1177/07067437211000636

**Published:** 2021-03-15

**Authors:** Jitender Sareen, Shay-Lee Bolton, Natalie Mota, Tracie O. Afifi, Murray W. Enns, Tamara Taillieu, Ashley Stewart-Tufescu, Renée El-Gabalawy, Ruth Ann Marrie, J. Don Richardson, Murray B. Stein, Charles N. Bernstein, James M. Bolton, Jianli Wang, Gordon J. G. Asmundson, James M. Thompson, Linda VanTil, Mary Beth MacLean, Sarvesh Logsetty

**Affiliations:** 1 Department of Psychiatry, University of Manitoba, Winnipeg, Manitoba, Canada; 2 Department of Clinical Health Psychology, University of Manitoba, Winnipeg, Manitoba, Canada; 3 Department of Community Health Sciences, University of Manitoba, Winnipeg, Manitoba, Canada; 4 Faculty of Social Work, University of Manitoba, Winnipeg, Manitoba, Canada; 5 Department of Anesthesia and Perioperative Medicine, University of Manitoba, Winnipeg, Manitoba, Canada; 6 Department of Internal Medicine, University of Manitoba, Winnipeg, Manitoba, Canada; 7 Department of Psychiatry, Western University, London, Ontario, Canada,; 8 St. Joseph’s OSI Clinic, London, Ontario, Canada; 9 Department of Psychiatry, UCSD School of Medicine, La Jolla, CA, USA; 10 Department of Internal Medicine, University of Manitoba, Winnipeg, Manitoba, Canada; 11 Institute of Mental Health Research, University of Ottawa, School of Epidemiology and Public Health, Faculty of Medicine, University of Ottawa, Ontario, Canada; 12 Department of Psychology, University of Regina, Saskatchewan, Canada; 13 Department of Public Health Sciences, Queens University, Kingston, Ontario, Canada; 14 Department of Family Medicine, Dalhousie University, Halifax, Nova Scotia, Canada; 15 Veterans Affairs Canada, Charlottetown, Prince Edward Island, Canada; 16 Department of Surgery, University of Manitoba, Winnipeg, Manitoba, Canada

**Keywords:** common mental disorders, military, veteran, epidemiology, longitudinal study, armed forces, prevalence, comorbidity

## Abstract

**Objective:**

The current study used the Canadian Armed Forces Members and Veterans Mental Health Follow-up Survey (CAFVMHS) to (1) examine the incidence and prevalence of mental disorders and (2) estimate the comorbidity of mental disorders over the follow-up period.

**Method:**

The CAFVMHS (2018) is a longitudinal study with two time points of assessment. The sample is comprised of 2,941 Canadian Forces members and veterans who participated in the 2002 Canadian Community Health Survey: Canadian Forces Supplement. The World Health Organization Composite International Diagnostic Interview (WHO-CIDI) was utilized to diagnose *Diagnostic and Statistical Manual*-IV post-traumatic stress disorder (PTSD), major depressive episode (MDE), generalized anxiety disorder, social anxiety disorder (SAD), and alcohol abuse and dependence. Self-report health professional diagnoses were assessed for attention deficit hyperactivity disorder (ADHD), mania, obsessive compulsive disorder (OCD), and personality disorder. We established weighted prevalence of mental disorders and examined the association between mental disorders using logistic regression.

**Results:**

In 2018, lifetime prevalence of any WHO-CIDI-based or self-reported mental disorder was 58.1%. Lifetime prevalence of any mood or anxiety disorder or PTSD was 54.0% in 2018. MDE (39.9%), SAD (25.7%), and PTSD (21.4%) were the most common mental disorders. There was a substantial increase in new onset or recurrence/persistence of mental disorders between the two measurement points (16-year assessment gap); 2002–2018 period prevalences were 43.5% for mood and anxiety disorder and 16.8% for alcohol abuse or dependence. The prevalence of self-reported ADHD, OCD, any personality disorder, and mania were 3.3%, 3.0%, 0.8%, and 0.8%, respectively. Comorbidity between mental disorders increased over the follow-up.

**Conclusions:**

This study demonstrates a high burden of mental disorders among a large Canadian military and veteran cohort. These findings underscore the importance of prevention and intervention strategies to reduce the burden of mental disorders and alcohol use disorders in these populations.

## Abstract (fr)

**Objectif:**

La présente étude a utilisé l’Enquête de suivi sur la santé mentale auprès des membres des Forces armées canadiennes et des ex-militaires (ESSMFACM) pour: 1) examiner l’incidence et la prévalence des troubles mentaux et 2) estimer la comorbidité des troubles mentaux durant la période du suivi.

**Méthodes:**

L’ESSMFACM (2018) est une étude longitudinale comportant une évaluation en deux points dans le temps. L’échantillon comprend 2 941 membres des Forces armées et des ex-militaires qui ont participé en 2002 à l’Enquête sur la santé dans les collectivités canadiennes - Forces canadiennes (ESCC-FC). L’entretien diagnostique international composite (EDIC) de l’ Organisation mondiale de la santé (OMS) a été utilisé pour diagnostiquer, conformément au Manuel diagnostique et statistique des troubles mentaux (DSM-IV), le trouble de stress post-traumatique (TSPT), l’épisode de dépression majeure (EDM), le trouble d’anxiété généralisée (TAG), le trouble d’anxiété sociale (TAS), l’abus d’alcool et la dépendance. Les diagnostics auto-vérifiés par des professionnels de la santé ont été évalués pour le trouble de déficit d’attention avec hyperactivité (TDAH), la manie, le trouble obsessionnel-compulsif (TOC), et le trouble de la personnalité. Nous avons établi une prévalence pondérée des troubles mentaux et examiné l’association entre les troubles mentaux à l’aide de la régression logistique.

**Résultats:**

En 2018, la prévalence de durée de vie de tout trouble mental basé sur l’EDIC de l’OMS ou auto-déclaré était de 58,1%. La prévalence de durée de vie de tout trouble de l’humeur ou anxieux, ou du TSPT était de 54,0% en 2018. L’EDM (39,9%), le TAS (25,7%), et le TSPT (21,4%) étaient les troubles mentaux les plus communs. Il y a eu une augmentation substantielle des nouveaux cas ou de la récurrence/persistance des troubles mentaux entre les deux points de mesure (écart d’évaluations de 16 ans); la prévalence de la période de 2002 à 2018 était de 43,5% pour le trouble de l’humeur et anxieux, et de 16,8% pour l’abus d’alcool ou la dépendance. La prévalence du TSPT et du TOC auto-déclarés, de tout trouble de la personnalité et de la manie était de 3,3%, 3,0%, 0,8%, et 0,8%, respectivement. La comorbidité entre les troubles mentaux augmentait au suivi.

**Conclusions:**

La présente étude démontre un fardeau élevé de troubles mentaux dans une vaste cohorte de militaires et d’ex-militaires canadiens. Ces résultats soulignent l’importance des stratégies de prévention et d’intervention en vue de réduire le fardeau des troubles mentaux et des troubles d’utilisation d’alcool dans ces populations.

There is increasing literature on the prevalence and impact of mental health problems and alcohol use disorders among members of the Canadian Armed Forces (CAF) and veterans.^1–3^ Canadian military members and veterans have been noted to have a higher prevalence of mental disorders than Canadian civilians.^4–7^ A complex interplay between preenlistment factors, deployment-related traumatic events, physical health problems (e.g., chronic pain), and difficulties in transition to civilian life following service contribute to the development of mental health problems and substance use disorders.^6^ Over the past 2 decades, Canadian military personnel have faced higher levels of traumatic experiences during deployment (e.g., Afghanistan) than during previous peacekeeping missions, potentially increasing risk of mental disorders.^1,3^ Transition to civilian life for military members has been demonstrated to be a vulnerable period associated with mental health problems and suicide risk^8,9^ due to several factors including financial stress, identity issues, psychosocial stress, and coping with physical health problems.^10–12^

Both CAF and Veterans Affairs Canada (VAC) have commissioned mental health and well-being surveys of military personnel and veterans.^5,13^ In 2002, a nationally representative survey of CAF personnel was conducted to estimate the prevalence of mental health problems among Regular and Reserve Force personnel (*N* = 8,441).^13^ The survey found 14.9% met criteria for a mood, anxiety, or alcohol use disorder during the past year in 2002.^13^ In 2013, a similarly designed survey (*N* = 8,161) was conducted and found a small but significant increase in past year prevalence of mental disorders (16.5%).^14^ This study also found that comorbidity of mental disorders (i.e., prevalence of multiple mental disorders) was higher in the 2013 survey compared to the 2002 survey.

Similarly, VAC in collaboration with Statistics Canada conducted the Life after Service Surveys in 2010, 2013, and 2016.^5^ Each survey included approximately 3,000 veterans. These cross-sectional surveys, using self-report diagnosis of mental health conditions, found that approximately 25% of veterans reported a past year mental health condition (i.e., mood, anxiety, or post-traumatic stress disorder [PTSD]). There was an observable increase in past year prevalence of mental health conditions from 2010 (25.8%) and 2013 (25.4%) surveys to the 2016 survey (30.3%).^5,6^

Although the above noted studies provide a “snapshot” of the burden of mental health problems among CAF personnel and veterans, there remain several important gaps in knowledge. The most important gap is that the mental health status of military members over time remains unknown. The proportion who develops a new onset or recurrent mental disorder or alcohol use disorder remains unclear. Transition to civilian life following military service is an important period of vulnerability, which is not captured by these surveys that were limited to either veterans or active duty members of the CAF but not both populations.^6^ Non-Canadian military and veteran studies have also been limited in estimating the prevalence of mental disorders because most have used self-report screening instruments that are not designed to make *Diagnostic and Statistical Manual* (*DSM*)-based diagnoses.^15–18^ Finally, little is known about the prevalence of certain disabling conditions in military and veteran samples that often present in clinical samples (e.g., personality disorders, attention deficit hyperactivity disorder [ADHD] and obsessive compulsive disorder [OCD]) but have rarely been covered in prior surveys.

To address these gaps in the literature, and in an effort to provide guidance for policymakers on current policy and strategies, it is necessary to clearly understand the burden of mental health in the CAF over time. To this end, we analyzed longitudinal data from 2,941 active and veteran Regular Force personnel who were part of the 2018 CAF Members and Veterans Mental Health Follow-up Survey (CAFVMHS) to:

1. delineate the incidence (2002 to 2018 period) and cumulative lifetime prevalence (as of 2018) of mental disorders;
2. estimate the past year prevalence of mental disorders in 2002 and 2018; and
3. determine the rate of comorbidity of mental disorders in 2002 and 2018.

## Method

### Sample

The CAFVMHS (2018) is a nationally representative two timepoints, longitudinal, cohort study that conducted in-person interviews of Regular Force service members and veterans who originally participated in the Canadian Community Health Survey on Mental Health and Wellbeing: Canadian Forces Supplement (CCHS-CFS) in 2002.^19^ The CCHS-CFS was a nationally representative sample of active duty CAF Regular Force personnel (*N* = 5,155). Of the 4,299 participants who were eligible for reinterview in 2018, 2,941 participated in the follow-up survey (response rate of 68% among those eligible for reinterview).^20^ Data were collected between January and May of 2018. Statistics Canada created longitudinal sampling weights to ensure representativeness of the 2018 sample to the original 2002 cohort and to account for nonresponse. In 2018, 87.8% of the sample was male, 65.5% were veterans, 60% were over 50 years old, and 82.7% were married.^21^ Additional details about the survey methodology and sociodemographic characteristics of the sample can be found elsewhere and on the Statistics Canada website.^19–21^

### Measures

#### Mental disorders

Mental disorders that had good reliability and validity through the World Health Organization Composite International Diagnostic Interview (WHO-CIDI) were measured using this instrument at 2002 and 2018. Mental disorders that did not have strong reliability and validity with the CIDI were measured by self-report questions.

#### CIDI-based mental disorders in 2002 and 2018

The WHO-CIDI is a fully structured diagnostic interview that was used to assess mental disorders according to *DSM*-IV criteria by trained lay interviewers. The validity and reliability of this instrument has been well-established.^1,22,23^ In 2002, lifetime and past year diagnoses were assessed for the following conditions—major depressive episode (MDE), PTSD, generalized anxiety disorder (GAD), panic disorder, and social anxiety disorder (SAD).^24^ Although PTSD is categorized as an anxiety disorder in *DSM*-IV, we analyzed and discussed this disorder separately because it is of special interest in this population, and *DSM*-5 has also separated PTSD from other anxiety disorders.^25,26^ Alcohol dependence was measured only for past year, not lifetime. Lifetime or past year alcohol abuse was not measured in 2002.^27^ In 2018, 16-year incidence and recurrence of the following mental disorders were measured between 2002 and 2018: MDE, panic disorder, SAD, GAD, PTSD, and alcohol abuse and dependence. During the 16-year follow-up period, people could have had a new onset of a mental disorder or a recurrence of a mental disorder. Individuals were only counted once in the estimate of 16-year incidence, persistence, and recurrence. Past year diagnoses were measured for all these conditions; however, presence of past 12-month PTSD diagnosis was calculated based on an algorithm of variables available in the data since a true past year diagnosis was not evaluated. This variable was created based on three criteria: (1) presence of a CIDI-based PTSD diagnosis in the 16-year follow-up, (2) responding “yes” to a single question that assessed whether the individual had PTSD-related reactions in the past 12 months, and (3) having at least 3 of the 7 PTSD symptoms that were assessed in a past year time frame. The algorithm for PTSD was created by Statistics Canada based on the US National Comorbidity Survey follow-up study.^28^ In 2002 and 2018, past year CIDI-based mental disorders were also categorized into 0, 1, 2, 3, or more disorders. These cut points have been used in previous literature to compare comorbidity rates over time.^14^

#### Self-reported mental disorders in 2018

Some mental disorders were assessed by self-report due to the poor reliability of the CIDI module for these disorders—namely personality disorders,^29^ ADHD,^30^ and OCD.^31^ These disorders were assessed in the chronic conditions section of the CAFVMHS by asking the respondent if they had a long-term health condition diagnosed by a health professional that had lasted or was expected to last 6 months or longer. The following mental disorders were assessed by self-report of diagnosis by a health professional: each of the 9 *DSM*-IV personality disorders, ADHD, mania, and OCD.

### Statistical Analyses

All analyses were conducted using STATA Version 16.^24^ Listwise deletion was used when missing data were present; this is standard practice since all variables examined had less than 5% missing values. Weights calculated by Statistics Canada were applied to all inferential analyses to ensure representativeness of all CAF members in 2002. Bootstrapping was used as a standard error estimation technique to account for the complex sampling design of the survey. Lifetime, past year, and since 2002 “new onset” prevalence rates and 95% confidence intervals were estimated at 2002 and 2018. Weighted cross-tabulations were used to estimate rates of comorbidity, and we conducted unadjusted logistic regressions to examine the comorbidity between the individual lifetime mental disorders at 2018. Due to methodological differences in the assessment of CIDI-based mental disorders and self-reported mental disorders, we only examined comorbidity between CIDI-based mental disorders.

## Results

Table 1 shows the lifetime prevalence of *DSM*-IV mental disorders in 2002, prevalence between 2002 and 2018, and cumulative lifetime mental disorders in 2018. In 2018, the cumulative lifetime prevalence of at least 1 mood or anxiety disorder was 54%. Among the disorders, MDE, SAD, and PTSD were the 3 most common conditions with 2018 lifetime prevalence of 40.0%, 25.8, and 22.0%. Between 2002 and 2018, the period prevalence of *DSM*-IV alcohol abuse or dependence was 16.8% (Table 1). Co-occurrence of mental disorders increased over time (3 or more disorders—2002: 3.1%, 2018: 21.9%). Among the self-reported health professional diagnosed mental disorders assessed in 2018, ADHD and OCD were found to have a prevalence of 3.3% and 3.0%, while mania and any personality disorder had a prevalence of 0.8%.

**Table 1.** Lifetime Prevalence of Mental Disorders in 2002, Incidence, Persistence or Recurrence between 2002 and 2018, and Cumulative Prevalence in 2018.

	2002 Sample Lifetime Prevalence at 2002		Incidence, Persistence, or Recurrence between 2002 and 2018		Cumulative Lifetime Prevalence at 2018	
	%^a^	95% CI	%	95% CI	%	95% CI
CIDI-based diagnoses						
Any mood or anxiety disorder	27.0	25.3 – 28.8	43.5	41.5 – 45.5	54.0	52.0 – 56.0
MDE	16.5	15.1 – 18.1	32.8	30.9 – 34.8	40.0	38.0 – 42.1
Any anxiety disorder	18.2	16.7 – 19.7	34.2	32.4 – 36.0	43.0	41.1 – 44.9
PTSD	7.0	6.1 – 8.1	17.6	16.1 – 19.1	22.0	20.4 – 23.8
GAD	4.8	4.0 – 5.7	15.2	13.8 – 16.8	18.3	16.7 – 19.9
Panic disorder	3.6	2.9 – 4.4	16.5	15.0 – 18.1	19.0	17.4 – 20.7
SAD	8.3	7.3 – 9.5	20.5	18.9 – 22.2	25.8	24.1 – 27.7
Level of Comorbidity^b^						
1 Disorder	16.3	14.9 – 17.8	15.3	13.9 – 16.9	19.1	17.5 – 20.8
2 Disorders	6.4	5.4 – 7.4	8.8	7.7 – 10.0	11.7	10.4 – 13.0
3+ Disorders	3.3	2.7 – 4.2	18.2	16.7 – 19.9	21.9	20.2 – 23.7
Alcohol abuse or dependence	—^c^	—	16.8	15.3 – 18.5	—	—
Alcohol abuse	—	—	10.8	9.6 – 12.2	—	—
Alcohol dependence	—	—	6.0	5.0 – 7.1	—	
Self-report diagnoses (lifetime)						
ADHD	—	—	—	—	3.3	2.6 – 4.2
OCD	—	—	—	—	3.0	2.4 – 3.9
Personality disorder	—	—	—	—	0.8	0.5 – 1.3
Mania	—	—	—	—	0.8	0.5 – 1.3
Any mental disorder (CIDI-based or self-report)					58.1	56.1 – 60.1

*Note*. MDE = major depressive episode, PTSD = post-traumatic stress disorder, GAD = generalized anxiety disorder, SAD = social anxiety disorder, ADHD = attention deficit hyperactivity disorder, OCD = obsessive compulsive disorder.

^a^ Weighted percentages, ^b^Not including alcohol use disorders, ^c^Cannot include due – measurement issues with CCHS-CFS 2002.

Table 2 demonstrates the past year prevalence of mental disorders in 2002 and 2018. All mood and anxiety disorders had a substantial increase in prevalence, often more than doubling. Past year alcohol dependence was the only condition that had a decrease in prevalence (2002: 3.8%; 2018: 2.3%). Comorbidity between mental disorders also increased over time (3 or more disorders—2002: 1.7%, 2018: 10.6%).

**Table 2.** Prevalence of Past-year World Health Organization Composite International Diagnostic Interview-based Mental Disorders in 2002 and 2018.

	2002 Sample Past Year		2018 Sample Past Year	
	%^a^	95% CI	%	95% CI
Any mood or anxiety disorder	11.8	10.5 – 13.1	27.6	25.8 – 29.5
MDE	7.6	6.5 – 8.8	19.5	18.0 – 21.2
Any anxiety disorder	7.1	6.1 – 8.3	25.6	24.9 – 27.4
PTSD	2.9	2.3 – 3.6	9.9	8.8 – 11.2
GAD	1.9	1.4 – 2.5	7.3	6.3 – 8.5
Panic disorder	1.8	1.3 – 2.5	11.0	9.6 – 12.4
SAD	3.3	2.6 – 4.1	13.6	12.2 – 15.1
Level of Comorbidity^b^				
1 Disorder	7.5	6.5 – 8.7	10.2	9.0 – 11.6
2 Disorders	2.1	1.6 – 2.7	5.6	4.7 – 6.6
3+ Disorders	1.7	1.2 – 2.4	10.6	9.3 – 12.0
Alcohol abuse or dependence	—^c^	—	3.8	3.0 – 4.7
Alcohol abuse	—	—	1.5	1.1 – 2.1
Alcohol dependence	3.8	3.0 – 4.8	2.3	1.7 – 3.0

*Note*. MDE = major depressive episode, PTSD = post-traumatic stress disorder, GAD = generalized anxiety disorder, SAD = social anxiety disorder.

^a^ Weighted percentages, ^b^Not including alcohol use disorders, ^c^Cannot include due – measurement issues with CCHS-CFS 2002.

Table 3 provides descriptive information about new onset and persistent/recurrent mood and anxiety disorders during the 16-year follow-up period. A clear pattern exists across all disorders; that the majority of cases had a new onset mental disorder. Approximately 26.3% had a new onset mood or anxiety disorder during the intervening period, while 17.4% had a persistent/recurrent mood or anxiety disorder.

**Table 3.** Breakdown of World Health Organization—Composite International Diagnostic Interview-based Mental Disorder Estimates according to Time of the Survey in 2018.

	None	Before 2002 Only	New Onset after 2002 Only	Persistent/Recurrent
	% (95% CI)^a^	% (95% CI)	% (95% CI)	% (95% CI)
Any mood or anxiety disorder	46.6 (44.5 – 48.6)	9.8 (8.6 – 11.0)	26.3 (24.5 – 28.1)	17.4 (15.9 – 19.0)
MDE	60.1 (58.1 – 62.1)	7.1 (6.1 – 8.1)	23.5 (21.7 – 25.3)	9.4 (8.3 – 10.6)
Any anxiety disorder	57.9 (55.9 – 59.9)	8.1 (7.0 – 9.3)	23.9 (22.1 – 25.7)	10.1 (9.0 – 11.4)
PTSD	78.6 (76.9 – 80.2)	4.1 (3.4 – 5.1)	14.5 (13.1 – 16.1)	2.7 (2.2 – 3.4)
GAD	81.8 (80.2 – 83.4)	3.0 (2.3 – 3.7)	13.3 (12.0 – 14.9)	1.9 (1.4 – 2.6)
Panic disorder	81.4 (79.7 – 83.0)	2.2 (1.7 – 2.9)	15.0 (13.6 – 16.6)	1.4 (0.9 – 2.0)
SAD	74.3 (72.5 – 76.0)	5.3 (4.5 – 6.2)	17.3 (15.8 – 19.0)	3.1 (2.5 – 3.9)

*Note*. MDE = major depressive episode, PTSD = post-traumatic stress disorder, GAD = generalized anxiety disorder, SAD = social anxiety disorder.

^a^ Weighted percentages.

Table 4 provides cross-tabulations and unadjusted odds ratios with respect to comorbidity between mental disorders. In the vast majority of cases, there was a significant association between the assessed mental disorders (i.e., odds ratios ranging between 7 and 11). GAD and MDE had the highest odds ratio for comorbidity (odds ratio = 11.65; 95% CI: 8.74 to 15.53). PTSD, GAD, panic disorder, and SAD all had high rates of comorbidity among those reporting a MDE (ranging from 73% to 83%).

**Table 4.** Comorbidity of Lifetime CIDI-based Mental Disorders with Odds Ratios (OR) and 95% Confidence Intervals (CI) for the Associations between Mental Disorders.

	Major Depressive Episode (MDE)		Post-traumatic Stress Disorder (PTSD)		Generalized Anxiety Disorder (GAD)		Panic Disorder		Social Anxiety Disorder (SAD)	
	%	OR (95% CI)	%	OR (95% CI)	%	OR (95% CI)	%	OR (95% CI)	%	OR (95% CI)
MDD	—	—	44.4^a^	9.91***(7.86 – 12.49)	38.4	11.65***(8.74 – 15.53)	36.8	7.51***(5.76 – 9.78)	48.4	7.62***(6.10 – 9.53)
PTSD	79.6	9.91*** (7.86 – 12.49)	—	—	46.0	7.42***(5.88 – 9.37)	50.8	9.75***(7.75 – 12.26)	58.8	7.46***(5.97 – 9.33)
GAD	83.3	11.65***(8.74 – 15.53)	55.4	7.42***(5.88 – 9.37)	—	—	50.5	7.53***(5.90 – 9.59)	65.1	9.19***(7.17 – 11.78)
Panic disorder	77.3	7.51***(5.76 – 9.78)	60.1	9.75***(7.75 – 12.26)	48.5	7.53***(5.90 – 9.59)	—	—	65.3	9.57***(7.50 – 12.20)
SAD	74.6	7.62***(6.10 – 9.53)	50.8	7.46***(5.97 – 9.33)	46.2	9.19***(7.17 – 11.78)	48.2	9.57***(7.50 – 12.20)	—	—

^a^ Of all people with MDD, 44.4% also had PTSD.

## Discussion

The current study provides important new information about the incidence and prevalence of mental and alcohol use disorders in a large prospective cohort of CAF active duty and veteran soldiers.

First, we found that 58.1% of the cohort met criteria for a mental disorder or a self-reported mental disorder at some point in their lifetime. The lifetime prevalence of 58.1% observed in the current study among military and veteran populations is much higher than the lifetime prevalence estimates of 12.7% to 47.4% found in general population surveys across 17 countries using CIDI based *DSM*-IV diagnoses (Canada was not included in this study).^32^ Most Canadian studies focus on past year prevalence; we were unable to find a study that had published lifetime prevalence of common mental disorders in Canada. To the best of our knowledge, only one study in the literature projected a lifetime prevalence of a comprehensive list of mental disorders among 671 US National Guard reservists.^33^ This study found a probable lifetime prevalence of 61% which is remarkably similar to the prevalence of 58% found in the current study.^33^ The high prevalence of mental disorders in our sample is likely due to the long time frame of assessments covering the person’s lifetime at 2002 and 16-year follow-up period. In addition to common mental disorders such as PTSD, mood, and anxiety disorders, we assessed other mental disorders that are not commonly assessed in military and veteran studies such as personality disorders, ADHD, and OCD.^27,34^ Finally, the high prevalence found in this cohort could be due to accumulation of mental disorders over time, and high levels of traumatic events including adverse childhood experiences and deployment-related traumatic events.^35,36^

Second, our study found a past year prevalence of mental disorder of 27.6% in 2018. This past year prevalence is substantially higher than the 16.1% past year mental disorder rate found in the 2013 CAF nationally representative sample and is higher than the Canadian General Population (∼20%).^5,7,37^ The current study’s finding is similar to the prevalence of mental disorders found in the Life after Service Survey of Canadian veterans (i.e., 25% to 30%).^5,6^ These findings are also remarkably consistent with a US study that examined a Veterans Health Administration primary care cohort (>4 million veterans) and found a quarter of the sample with at least one mental disorder diagnosis corresponding to >1.15 million veterans.^38^ A slightly lower prevalence (22%) was found in a large UK military cohort (*N* = 8,093) that was engaged in the Afghanistan and Iraq mission.^39^

Third, we found that the onset of the majority of mental disorders occurred between 2002 and 2018 compared to the pre-2002 period. There are several explanations for these findings. One possibility is that the recall period at 2018 was 16 years, while the 2002 survey had a longer recall period that may be associated with increased recall error. Another hypothesis is that there was greater exposure to traumatic events during deployments between 2002 and 2018 than prior to 2002.^1,7,40^ During 2002 to 2018 period, deployment missions to Afghanistan were associated with much higher levels of combat and life-threatening traumatic events than pre-2002 deployments.^1^ Additionally, a large percentage of the survey participants had transitioned from active duty to veteran during this time frame. Previous work suggests transition to civilian life is a stressful period that is associated with mental disorder onset.^2,6^ The increases in rates of mental disorders over time are consistent with previous Canadian^5–7^ and US literature.^41^ Finally, a reduction in mental health stigma and expansion of mental health services for veterans through the Operational Stress Injuries clinics across Canada may also explain the increase in period prevalence between 2002 and 2018.

Fourth, there were some important findings with respect to the prevalence of specific mental disorders. In 2018, MDE, PTSD, and SAD were the most prevalent lifetime mental disorders in the sample—40.0%, 25.8%, 22.0%, respectively (Table 1). These are common conditions associated with traumatic and stressful life experiences.^42^ We were surprised that a large number of new onset SAD cases occurred during the follow-up period because this disorder usually has an onset during adolescence. There is very limited epidemiological data on the prevalence of SAD among military and veterans. Our group found a lifetime prevalence of SAD of 8.2% in the 2002 CAF sample that included Regular Force and Reserve members.^43^ Small clinical samples of US Vietnam veterans have found the prevalence of SAD and PTSD of 32% which is similar to our study.^33^ Previous research with Vietnam veterans clinical samples has found a strong link between PTSD, depressive symptoms, and social withdrawal.^44^ It is possible that the high prevalence of SAD may be due to comorbidity with PTSD and depression that might be leading to social avoidance and disability. Among self-reported mental disorders, we found that ADHD and OCD were common in this sample at approximately 3% each, while personality disorder was less than 1.0%. The US National Guard study projected a lifetime prevalence of OCD as 3.7% which is quite similar to the findings in our study.^44^ We were unable to find other epidemiological studies in active military or veterans that estimated the prevalence of ADHD and personality disorders. The prevalence of a personality disorder in general population samples in the UK and US has ranged between 9% and 10%.^45,46^ Any personality disorder prevalence was 0.8% in our study and is likely an underestimate because these diagnoses were based on self-reported mental disorders rather than a structured diagnostic interview.^47^ However, it is possible that the prevalence of personality disorders is lower than in the general population because of the “Healthy Warrior” effect (i.e., military members are healthier than civilian counterparts).^39^ The 3.0% prevalence of ADHD in the current study was similar to the 2.8% found in civilian populations across multiple countries using a structured diagnostic interview.^48^ Since ADHD was assessed by self-report, it is possible that this prevalence is also an underestimate.

Fifth, we found that the past year prevalence of alcohol dependence decreased between 2002 (3.8%) and 2018 (2.3%). A UK military cohort also noted a decrease in prevalence of alcohol misuse.^34^ A systematic review of substance use disorders among US veterans between 1994 and 2014 found a decreasing prevalence of substance use disorders.^49^ This trend in decreasing prevalence of alcohol use disorders among veterans across different countries may be related to greater awareness of the harmful effects of alcohol misuse. Nonetheless, the burden of alcohol use disorders during the 2002to 2018 period remained high at 16% period prevalence. These high numbers underscore the need for continuing efforts with respect to prevention and treatment of alcohol use disorders.

Sixth, the comorbidity of mental disorders increased over the follow-up period. The past year prevalence of 3 or more disorders increased from 3.1% in 2002 and 21.9% in 2018. This is likely due to a long period of time for mental disorders and accumulation of mental disorders as the individual ages.^13,50^ Among the disorders assessed, PTSD had the highest rate of comorbidity with mood and anxiety disorders (Table 4). It is also possible that stigma and other barriers to care pose impediments in care-seeking which contribute to the development of comorbidity.^27,51^ Previous work has shown that attitudinal barriers (e.g., “afraid of what others would think”) are more prevalent than structural barriers (e.g., “could not afford to pay”) to mental health care among military members and veterans.^20^

### Strengths and Limitations

The results of the study need to be considered in the context of its several strengths and limitations. The main strength of this survey is that the same individual is followed up after a 16-year period. Other notable strengths include large sample, high response rate, use of structured interviews to evaluate *DSM*-IV diagnoses for the majority of mental disorders, and assessment of mental health status both during military service and after transition to civilian life for some participants. Limitations of the study include CIDI-based diagnoses that may not match the accuracy of clinician-based diagnoses, the creation of an algorithm to assess past year PTSD, and the use of self-reported health professional diagnoses for some mental disorders (e.g., ADHD) which may underestimate actual prevalence.^20^ Although the analysis included weighting for nonresponse, it is possible that findings could be affected by selection bias related to differences between respondents and nonrespondents (e.g., in rates of mental disorders). However, Bolton et al.^34,52^ found that baseline mental disorders and many other factors investigated were not associated with attrition in this longitudinal survey.^53,54^ Both time points of the survey used the CIDI-based *DSM*-IV diagnostic criteria. Changes to the diagnostic criteria between *DSM*-IV and *DSM*-5 may impact reported findings. Future investigations using *DSM*-5 criteria are needed. Additionally, the long period of recall at both time points (i.e., lifetime at baseline and 16 years at follow-up) could be associated with recall errors. Evidence from previous studies suggest that lifetime estimates are associated with under reporting of previous depressive episodes and suggest that the current study’s lifetime and 16-year estimates of mental disorders might be lower than the true burden of mental illness.^34,52^ The current study presents the prevalence of mental disorders in the 2018 sample that includes both active-duty military personnel and veterans because we wanted to examine the whole cohort that was representative of the 2002 Nationally representative sample. Future studies will stratify the 2018 sample by active military and veteran status to estimate prevalence of correlates across these important subgroups. Finally, since the study only included exclusively 2002 Regular Force personnel, current findings are not generalizable to Reservists or to particular subgroups within this population (e.g., female CAF personnel only, young recruits).

In conclusion, this study provides new information about the high lifetime (∼6 of 10) and past year prevalence (∼1 of 4) of mental disorders in the military and veteran sample. The study also shows that in this cohort there was a substantial increase in prevalence of mental disorders as military members age and transition to civilian life. Comorbidity of mental disorders increased over the 16-year follow-up period suggesting a need for early interventions to prevent occurrence of comorbidity. Further studies will examine the predictors of these conditions and will inform policy makers on prevention and intervention strategies.^53,54^
